# Gut-lung axis in allergic asthma: microbiota-driven immune dysregulation and therapeutic strategies

**DOI:** 10.3389/fphar.2025.1617546

**Published:** 2025-07-31

**Authors:** Jian Lv, Yu Zhang, Shuang Liu, Ruoyu Wang, Jianan Zhao

**Affiliations:** ^1^ Graduate School, Heilongjiang University of Chinese Medicine, Harbin, China; ^2^ The Affiliated Traditional Chinese Medicine Hospital, Guangzhou Medical University, Guangzhou, China; ^3^ Department of Rheumatology, Guanghua Hospital Affiliated to Shanghai University of Traditional Chinese Medicine, Shanghai University of Traditional Chinese Medicine, Shanghai, China

**Keywords:** gut microbiota, SCFAs, allergic asthma, gut-lung axis, immune regulation, probiotics

## Abstract

**Background:**

Allergic asthma, a chronic respiratory disorder, is intricately linked to gut microbiota dysbiosis and metabolite perturbations through the gut-lung axis.

**Objective:**

This review the relationship between microbial immune crosstalk and the onset of asthma, with the aim of determining the mechanism by which gut microbiota drives the onset of asthma and providing evidence for therapeutic interventions.

**Methods:**

Literature search was conducted on PubMed using keywords (“gut microbiota” or “gut microbiota” or “gut microbiota metabolites” or “lung gut axis”), (“allergic asthma” or “asthma”), and (“immune regulation”), without date restrictions. Including peer-reviewed studies on human/animal models, articles that do not meet the requirements are excluded.

**Result:**

Microbial imbalance in asthma patients—marked by reduced α-diversity, depletion of immunomodulatory taxa (e.g., Bifidobacterium, Faecalibacterium), and enrichment of pathobionts—disrupts short-chain fatty acid (SCFA) and tryptophan metabolism, skewing Th17/Treg balance toward Th2-dominated inflammation and airway hyperresponsiveness. SCFAs, particularly butyrate, activate GPR41/43 receptors and inhibit histone deacetylases (HDACs), enhancing Treg differentiation while suppressing Th2/Th17 responses. Tryptophan metabolites, such as indole derivatives, alleviate pulmonary inflammation via aryl hydrocarbon receptor (AhR)-dependent IL-22 production. Clinically, diminished SCFA levels correlate with impaired immune tolerance and airway remodeling, while probiotics (*Lactobacillus*, Bifidobacterium), prebiotics, and high-fiber diets restore microbial equilibrium, attenuating asthma severity.

**Conclusion:**

Future research must integrate multi-omics data to delineate strain-specific functions, host-microbe interactions, and individualized responses influenced by genetics, diet, and environmental factors. This review underscores the gut microbiota’s dual role as a biomarker and therapeutic target, advocating for microbiota-directed strategies in asthma prevention and precision medicine.

## 1 Introduction

Allergic asthma, a prevalent chronic respiratory disorder, imposes a substantial global health burden. Epidemiological data reveal that 60%–80% of asthma cases exhibit allergic phenotypes, constituting the predominant disease subtype (Zhang et al., 2025). Notably, geographic disparities exist in prevalence trends: high-income countries demonstrate plateauing incidence rates of allergic disorders, whereas low- and middle-income nations face escalating trends, potentially reflecting environmental and socioeconomic determinants (Genuneit and Standl, 2022). Clinically, this condition manifests through characteristic airway hyperresponsiveness, episodic dyspnea, nonproductive cough, and chest constriction, with symptom severity correlating with allergen exposure levels (Shah and Newcomb, 2018). Importantly, 40%–60% of patients present with allergic multimorbidity, particularly concurrent allergic rhinitis and atopic dermatitis, which synergistically exacerbate disease progression and substantially impair quality of life metrics (Humbert et al., 2019).

The gut microbiota, comprising a complex ecosystem of commensal microorganisms, exerts systemic immunomodulatory effects through bidirectional gut-lung axis communication. With estimated microbial densities exceeding 10^14^ organisms and compositionally diverse taxonomy, this microbial consortium critically regulates host physiological homeostasis, including nutrient assimilation, xenobiotic metabolism, and intestinal epithelial barrier fortification (Yang and Chun, 2021). Mounting evidence from multi-omics studies implicates microbial imbalance in the pathogenesis of diverse disease states, spanning metabolic syndrome, autoimmune disorders, and neuropsychiatric conditions (Ding et al., 2024). Mechanistically, microbial-derived metabolites such as short-chain fatty acids (SCFAs) serve as key immunoregulatory mediators, modulating T-cell differentiation pathways and attenuating systemic inflammation through G protein-coupled receptor interactions (Li et al., 2022a). We hypothesize that gut microbiota modulation can recalibrate Th2/Th17-Treg imbalances in allergic asthma.

Emerging evidence has elucidated critical cross-talk between gut microbial communities and pulmonary pathophysiology through the gut-lung axis—a bidirectional immunoregulatory network involving microbial metabolites and immune cell trafficking. Contemporary research demonstrates that taxonomic alterations and functional perturbations in gut microbiota (microbial imbalance) significantly impact respiratory disease trajectories, particularly in COPD and allergic asthma exacerbations, as evidenced by longitudinal clinical cohort studies (Shi et al., 2021). Mechanistic investigations reveal that depleted microbial diversity compromises pulmonary immune homeostasis, predisposing hosts to enhanced type 2 inflammation and impaired antiviral defense mechanisms, as substantiated by preclinical models of allergic airway disease (Neag et al., 2022). Microbial-derived metabolites, including but not limited to SCFAs, exert distal immunomodulatory effects through systemic circulation and vagus nerve signaling—notably regulating Treg cell differentiation and suppressing neutrophilic infiltration in bronchial tissues via G protein-coupled receptor (GPCR) signaling pathways (Alswat, 2024). These mechanistic insights are revolutionizing our understanding of asthma pathogenesis, positioning microbiota-targeted interventions (e.g., probiotics, postbiotics) as promising disease-modifying approaches in precision allergology.

## 2 Methods

### 2.1 Search strategy

To identify published studies, we conducted a comprehensive search of PubMed and Embase databases, covering records up to January 2025. Our search strategy includes the following keyword sets: (“gut microbiota” or “gut microbiota” or “gut microbiota metabolites” or “lung gut axis”), (“allergic asthma” or “asthma”), (“immune regulation”). We only search for English publications. Preliminary screening is conducted using search engines provided by various databases.

### 2.2 Data extraction and synthesis

Before reading the full text of a given paper, we manually select references related to the topic using Excel software. Finally, all included are peer-reviewed articles related to the topic. During the process of writing the paper, one author is responsible for data extraction. Subsequently, other authors conducted cross checks on the extracted data to maintain its integrity and reliability.

## 3 Gut microbiota composition in allergic asthma

The gut microbiota in healthy individuals forms a phylogenetically complex ecosystem comprising bacteria, archaea, fungi, and viruses, with bacterial dominance primarily observed in four phyla: Firmicutes, Bacteroidetes (including genera like Prevotella), Actinobacteria (exemplified by Bifidobacterium species), and Clostridia (particularly the butyrate-producing species Faecalibacterium prausnitzii). This microbial consortium demonstrates substantial α-diversity (intra-individual species richness) and functional redundancy, enabling robust ecosystem stability that facilitates immune tolerance, epithelial barrier maintenance, and metabolic homeostasis through mechanisms including short-chain fatty acid (SCFA) biosynthesis (notably butyrate) and essential vitamin production (Liu et al., 2024; Mirmohammadali and Rosenkranz, 2023). Bidirectional communication with the central nervous system further establishes the microbiota’s role in regulating mood and cognitive functions through neuroendocrine, immune, and neural pathways, a relationship termed the gut-brain axis (Alves et al., 2024; Salami, 2021). These multifunctional interactions underscore the necessity of preserving microbial compositional integrity for systemic health maintenance.

Microbial diversity serves as a key biomarker for health status and pathological susceptibility. Epidemiological evidence associates reduced gut microbiota diversity with increased incidence of metabolic disorders (obesity, diabetes), cardiovascular pathologies, and immune-mediated conditions including allergic diseases (Guo et al., 2020; Shi et al., 2024). The diversity-immune function nexus manifests through microbial regulation of immune cell differentiation and inflammatory responses, where high diversity correlates with enhanced immune homeostasis and reduced chronic inflammation risks (Liu et al., 2024; Sottas et al., 2021). This immunological modulation, coupled with direct metabolic contributions, positions microbiota diversity preservation as a critical factor not merely for gastrointestinal health, but for comprehensive disease prevention strategies spanning multiple physiological systems.

Emerging evidence highlights significant compositional disparities between the gut microbiota of allergic asthma patients and healthy populations. Ke et al. demonstrated an inverse correlation between childhood gut microbiota diversity and susceptibility to asthma/allergic conditions, implicating depauperate microbiota as a potential risk amplifier (Ke et al., 2021). Asthma patients characteristically exhibit diminished α-diversity, a feature mechanistically linked to dysregulated immune homeostasis (Heinrich et al., 2023; Hsu et al., 2021). Concurrently, these individuals display depletion of immunomodulatory taxa like Bifidobacterium and *Lactobacillus*, alongside pathobiont enrichment such as *Escherichia* coli—a microbial signature that may perpetuate inflammatory cascades (Heinrich et al., 2023; Li et al., 2022b). Mechanistically, early-life microbial colonization patterns exert long-term immunological consequences, exemplified by *Clostridium difficile* establishment at 1 month predicting asthma development by age 6–7 years (van Nimwegen et al., 2011). Parallel microbial imbalance extends to respiratory ecosystems, where asthma severity correlates positively with reduced bacterial diversity and elevated proteobacterial abundance in the airway microbiome (Carr et al., 2019). Age-stratified analyses further reveal distinct microbiota configurations between asthmatic and non-asthmatic cohorts across developmental stages (Lee et al., 2019). Exogenous modifiers including antibiotic exposure and dietary patterns potentially exacerbate these ecological perturbations, creating feedforward loops that may intensify symptomatology. Therapeutic modulation through targeted probiotics or precision nutrition emerges as a promising strategy to restore microbial equilibrium, with clinical studies suggesting concomitant improvements in both immunological parameters and quality-of-life metrics (Liao et al., 2022; Ozerskaia et al., 2021). This review systematically delineates gut microbiota alterations in allergic asthma (summarized in Table 1), providing a framework to understand their pathogenic contributions and therapeutic potential.

**TABLE 1:** The correlation between gut microbiota and allergic asthma.

Reference	Bacterial genus	Type of study	Material studied/model	Results of allergic compared with non-allergic subjects
van Nimwegen et al. (2011)	*Clostridium difficile*	Clinical	Fecal samples	Colonisation with C.difficile at 1 month of age wasassociated with increased risk of asthma (OR: 2.06; 1.16–3.64) at 6 years of age
Sagar et al. (2014)	Bifidobacterium breve M-16 V, *Lactobacillus* rhamnosus NutRes1	*In-vivo*	Mouse model	Bifidobacterium brevis M-16 V and *Lactobacillus* rhamnosus NutRes1 have strong anti-inflammatory effects and alleviate asthma by regulating T cell responses
Stiemsma et al. (2016)	Lachnospira, *Clostridium* neonatale	Clinical	Fecal samples	Lachnospira decreases and *Clostridium* neonatale increases in asthmatic children 3 months after birth
Fujimura et al. (2016)	Bifidobacterium, Akkermansia and Faecalibacterium, *Candida* and Rhodotorula	Clinical	Fecal samples	Children at high risk of asthma have lower relative abundance of Bifidobacterium, Akkermansia, and Faecalibacterium, while specific fungi such as *Candida* and Rhodotorula cerevisiae have higher relative abundance
Hevia et al. (2016)	Bifidobacterium	Clinical	Fecal samples	Long term asthma patients have lower levels of bifidobacteria
Demirci et al. (2019)	Akkermansia muciniphila, Faecalibacterium prausnitzii	Clinical	Fecal samples	Compared with the healthy control group, A. muciniphila (5.45 ± 0.004 vs. 6.74 ± 0.01) and F. prausnitzii (5.71 ± 0.002 vs. 7.28 ± 0.009) were both reduced in the allergic asthma group
Hu et al. (2021b)	Faecalibacterium prausnitzii	*In-vivo*	Mouse model	Faecalibacterium prausnitzii alleviates symptoms of allergic asthma in mice by improving gut microbiota dysbiosis
Gao et al. (2021)	Prevotella bacteria	Clinical	Fecal samples	Maternal carriage of Prevotella copri during pregnancy decreases the offspring’s risk of asthma via production of succinate
Versi et al. (2023)	*Haemophilus* influenzae	Clinical	sputum	In neutrophilic asthma, there was greater abundance of *Haemophilus* influenzae and *Moraxella catarrhalis*
Mousavian et al. (2024)	Bifidobacterium, Lachnospira, Roseburia and Flavonifractor	Clinical	Fecal samples	Lower levels of bifidobacteria and Lachnospira are associated with a higher risk of allergies. In contrast, higher levels of Roseburia and Flavonifractor are associated with lower allergy risk

## 4 Biological activity of gut microbial metabolites

The gut microbiota-derived metabolites exhibit significant biological activities through intricate biosynthetic pathways and host-microbe interactions. Short-chain fatty acids (SCFAs), principal microbial fermentation products of dietary fibers, are synthesized via carbohydrate-active enzymes expressed by commensal bacteria. These ≤6-carbon molecules–predominantly acetate, propionate, and butyrate–exist in strictly regulated colonic ratios (Fernandes et al., 2014). Acetate production predominates through the Wood-Ljungdahl pathway in acetogenic bacteria, demonstrating superior metabolic efficiency compared to other SCFAs (Miller and Wolin, 1996). Propionate biosynthesis occurs via three distinct routes: Bacteroidetes species preferentially employ the succinate pathway (Reichardt et al., 2014), while Firmicutes utilize acrylate and propanediol pathways, particularly when metabolizing pentoses/hexoses (Macfarlane and Macfarlane, 2003). Butyrogenesis is specialized to select Firmicutes taxa expressing butyryl-CoA:acetate CoA-transferase, with Faecalibacterium prausnitzii being a key producer (Louis et al., 2010). SCFAs are key factors linking gut microbial imbalance with allergic airway diseases. Clinically relevant SCFA deficiencies are observed in allergic rhinitis patients and infants predisposed to later asthma/wheezing development (Zhou et al., 2021), establishing these metabolites as critical mediators in allergic airway pathogenesis (Cheng et al., 2022; Roduit et al., 2019). Some scholars speculate that low levels of butyrate may be associated with increased severity in asthma patients.

Tryptophan metabolism represents another pivotal microbial-host interaction axis. While dietary tryptophan is primarily absorbed intestinally for protein synthesis, colonic microbiota extensively catabolize residual tryptophan through multiple pathways. Direct bacterial conversion yields immunomodulatory indole derivatives (indole, IE, IPA, ILA) via tryptophanase-expressing species like *Escherichia coli* and *Proteus* vulgaris (Lee and Lee, 2010; Palusiak, 2013; Smith, 1897). Concurrently, microbial regulation of host tryptophan metabolism occurs through serotonin synthesis and kynurenine pathway modulation(Roager and Licht, 2018). These metabolites demonstrate dual neuroimmune regulatory capacity: indole derivatives activate aryl hydrocarbon receptor (AhR) signaling to promote anti-inflammatory cytokine production (Brown et al., 2022; Zhao et al., 2024), while kynurenine accumulation correlates with chronic inflammatory and neuropsychiatric disorders (Basnet et al., 2023; Diether et al., 2023).

The gut-lung axis operationalizes these metabolites through systemic immunomodulation. SCFAs mitigate allergic airway inflammation via GPR41/43-mediated suppression of Th2 responses and HDAC inhibition-induced Treg cell expansion, as evidenced by their therapeutic efficacy in murine asthma models (Bloor and Mitchell, 2021; Zhang et al., 2025). Clinical translation potential is suggested by probiotic interventions restoring SCFA levels and improving respiratory outcomes (Hu et al., 2021b; Thorburn et al., 2015). Similarly, tryptophan metabolites regulate pulmonary immunity through AhR-dependent IL-22 production and Th17/ILC3 modulation, with microbial imbalance-induced kynurenine/SCFA imbalances exacerbating airway hyperreactivity (Basnet et al., 2023; Padhi et al., 2024). These mechanistic insights position microbial metabolite modulation as a promising therapeutic strategy for allergic asthma and related airway pathologies, bridging microbial ecology with clinical immunology through targeted microbiome engineering approaches.

## 5 The interaction between gut microbiota and immune system

**FIGURE 1:**
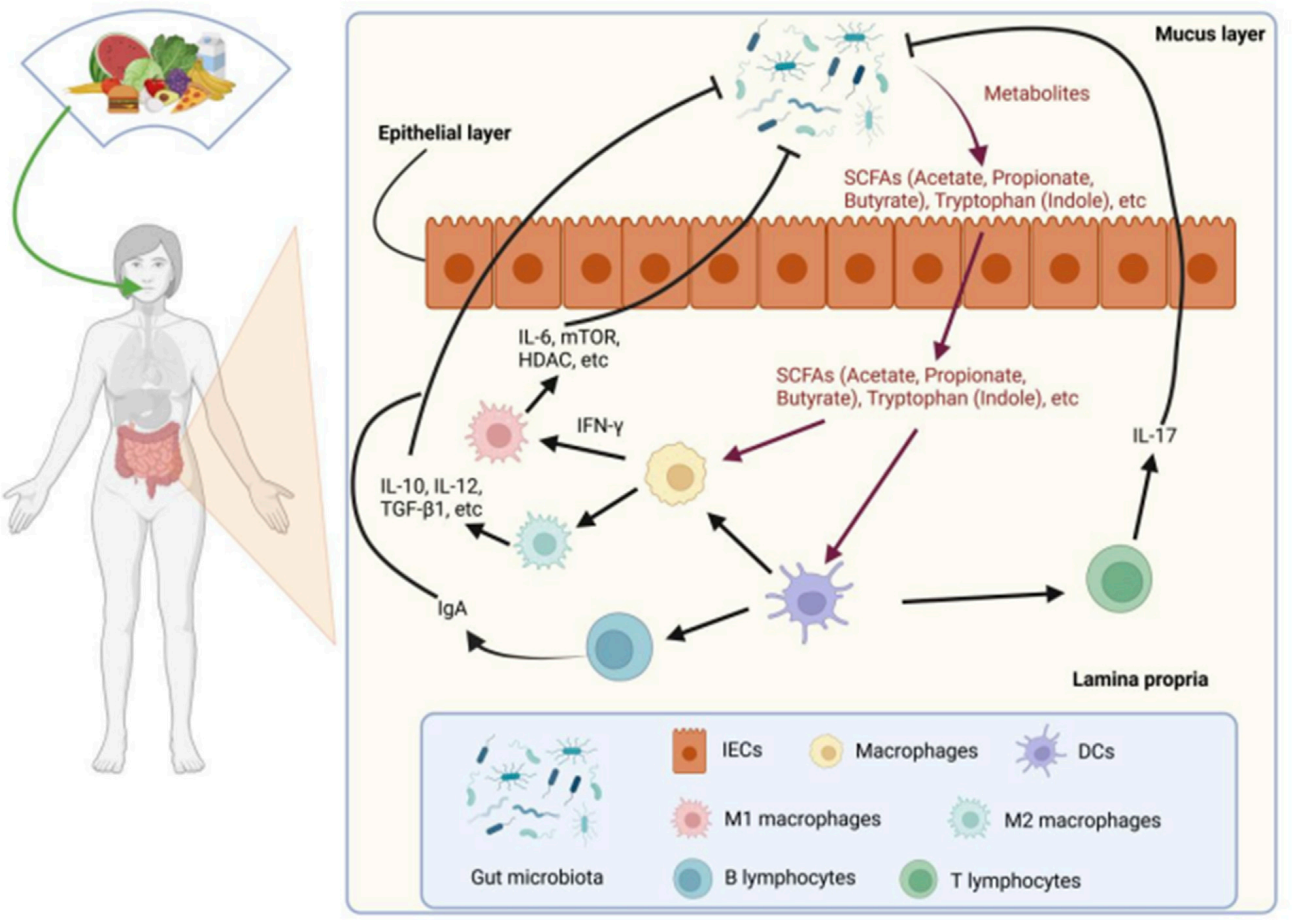
The interaction between gut microbiota and gut immune system. Metabolites of gut microbiota, such as SCFAs, can activate DCs and macrophages. Stimulation of DCs can activate B lymphocytes and T lymphocytes. The activation of M1 subtype macrophages, M2 subtype macrophages, B lymphocytes, and T lymphocytes can inhibit gut microbiota by releasing factors such as IL6, IL-10, IL12, IL17, and TGF - β. SCFAs, Short-chain fatty acids; DCs, Dendritic Cells.

The dynamic interplay between gut microbiota and the host immune system represents a fundamental axis in maintaining physiological homeostasis (Chen et al., 2022). Through multifaceted mechanisms—including immune cell modulation, metabolite production, and intestinal barrier maintenance—the microbiota directly shapes systemic immune competence. Microbial imbalance is increasingly implicated in immune-mediated pathologies such as autoimmune disorders, allergies, and inflammatory bowel diseases (Al-Rashidi, 2022; Yoo et al., 2020). Key findings from investigations into these interactions are systematically summarized in Table 2 and Figure 1.

**TABLE 2:** The characteristics and functions of metabolites in the main gut microbiota.

Metabolites of gut microbiota	Examples	Receptors	Effector cell	Cytokine	Specific function	Reference
SCFAs	Formic acid Acetate Propionate Butyrate Valeric acid	GPR41 (Yang et al., 2020) GPR43 (Sun et al., 2018) GPR109a (Thangaraju et al., 2009)	T cell	IL-10, GPCRs, STAT3, mTOR, HDAC, Foxp3, IL-6/STAT3/IL-17, acetyl CoA/mTOR, RORγt, mitoROS/Foxp3, NR4A1	Inducing differentiation of CD4^+^T cells	Arpaia et al. (2013), Balmer et al. (2016), Chen et al. (2019), Dupraz et al. (2021), Furusawa et al. (2013), Hang et al. (2019), Kespohl et al. (2017), Kibbie et al. (2021), Li et al. (2021), Luu et al. (2018), Park et al. (2015), Rangan and Mondino (2022), Shiratori et al. (2023), Smith et al. (2013), Song et al. (2023), Sun et al. (2018), Zhou et al. (2018)
					Enhanced memory transfer of CD8^+^ T cells	
			B cell	BCR, TLRs, IL-10	Increase acetyl CoA to promote energy and antibody production	Föh et al., 2022; Kim (2016), Kim et al. (2021)
			DCs	IL-6, IL-12 p40, IL-10, GPR109A, NF-κB, cAMP-PKA, TGR5, HDAC, TGF-β	Affect the differentiation of DCs generated from human monocytes and can inhibit T cell proliferation	Hu et al. (2021a), Hu et al. (2022), Isobe et al. (2020), Kaisar et al. (2017), Nastasi et al. (2015), Wu et al. (2017)
			Macrophages	HDAC3, mTOR, PhoP K102, NO,WNT/ERK, NF-κB, IFN-β	Modulate the function of intestinal macrophages	Chang et al. (2014), Liang et al. (2022), Park et al. (2007), Park et al. (2019), Schulthess et al. (2019), Tang et al. (2023)
Tryptophan metabolites	Indole IPA IAA	PXR (Venkatesh et al., 2014) AhR (Hubbard et al., 2015)	T cell	IL-17	Promote the production of T-regs cells while inhibiting the development of Th-17	Cervantes-Barragan et al. (2017), Rouse et al. (2013), Singh et al. (2016), Wilck et al. (2017)
					Activated the aryl-hydrocarbon receptor in CD4^+^ T cells, and differentiation into DP IELs	
			DCs	IL-10, IFN-γ, STAT3	Regulating cell differentiation and exerting anti-inflammatory effects	Aoki et al. (2018), Hwang et al. (2018)
			Macrophages	IL-10, IL-12, TGF-β1, TNFα, IL-23, IL-6, IFN-γ	Activate macrophages and downregulate pro-inflammatory cytokines	Huang et al. (2022b), Mohammadi et al. (2018)
			B cell	IL-35^+^, TLR4	IAA promotes the production of IL-35+B cells	Su et al. (2022)

Abbreviations: AhR, aryl hydrocarbon receptor; PXR, Retinoid X receptor; IPA, indolepropionic acid; IAA, 3-idoleacetic acid; HDAC3,histone deacetylase 3; NMDA, N-methyl-D-aspartate receptors; PXR, pregnane X receptor; SCFAs, short-chain fatty acids; DCs: Dendritic Cells.

### 5.1 T cell regulation

Gut microbiota critically influences T cell ontogeny and polarization. Microbial-derived short-chain fatty acids (SCFAs), generated through dietary fiber fermentation, drive the differentiation of regulatory T cells (Tregs), a process essential for immune tolerance and prevention of autoimmunity (Wang et al., 2024; Yoo et al., 2020). SCFAs bind to G protein-coupled receptors (e.g., GPR41, GPR43) on intestinal epithelial and immune cells, enhancing barrier integrity, suppressing inflammation, and modulating T cell subsets. Butyrate, for instance, serves dual roles as a primary energy source for colonocytes and an anti-inflammatory mediator via inhibition of pro-inflammatory cytokine production, as demonstrated by Chen et al. (2018). Importantly, SCFAs promote Treg expansion while suppressing pro-inflammatory Th17 cell activation, thereby exerting protective effects in allergic and autoimmune contexts (Gong et al., 2023; Yang and Cong, 2021). Furthermore, specific microbial taxa enhance CD8^+^ T cell cytotoxicity, as evidenced by Baruch et al. Research by Baruch et al. (2021), who reported improved anti-tumor immunity through microbiota-driven CD8^+^ T cell priming. These findings underscore the microbiota’s role as a rheostat for T cell homeostasis, where microbial imbalance may disrupt effector-regulatory balances, predisposing to immune dysregulation (Wang and Gong, 2022).

### 5.2 Cytokine-mediated crosstalk

Microbial-immune crosstalk is further mediated through cytokine networks. Gut commensals stimulate epithelial and immune cells to secrete cytokines that orchestrate local and systemic immunity. For example, select taxa induce anti-inflammatory IL-10 production, counteracting inflammation, while others trigger pro-inflammatory cytokines like TNF-α and IFN-γ, exacerbating conditions such as inflammatory bowel disease (Saini et al., 2022). Microbial imbalance disrupts this cytokine equilibrium, skewing responses toward pathogenic inflammation or immunosuppression (Guo et al., 2021; Hou et al., 2022). This regulatory nexus highlights the microbiota’s capacity to calibrate immune activation thresholds through cytokine signaling.

### 5.3 Barrier-immune interactions

The intestinal barrier—comprising mucus layers, epithelial tight junctions, and mucosal immune cells—acts as a frontline defense against luminal pathogens (Ulluwishewa et al., 2022). Gut microbiota fortify this barrier by stimulating epithelial cell proliferation, mucus secretion, and tight junction protein expression (e.g., occludin, claudins) (Huang et al., 2022a; Yoo et al., 2020). Conversely, microbial imbalance impairs barrier function, precipitating “leaky gut” syndrome, wherein bacterial translocation incites systemic inflammation and immune activation (Stepanova and Aherne, 2024; Terciolo et al., 2019). Thus, microbiota-barrier interactions are pivotal not only for intestinal health but also for preventing extra-intestinal immune pathologies. Collectively, these mechanisms illustrate the microbiota’s indispensable role in immune system education and regulation, positioning microbial modulation as a strategic target for immune-related disease management.

### 5.4 Other immune components

Short-chain fatty acids (SCFAs) enhance acetyl-CoA production, promoting antibody synthesis and IL-10 secretion (Luu et al., 2019). Tryptophan metabolites (e.g., indole-3-acetic acid) induce IL-35^+^ B cells via TLR4 signaling (Tomii et al., 2023). SCFAs inhibit pro-inflammatory cytokine (IL-6, IL-12) release from DCs via GPR109A and HDAC suppression, skewing T cell differentiation toward tolerance (Kleuskens et al., 2022). Gut microbial metabolites (e.g., propionate) modulate macrophage phagocytosis and anti-inflammatory function via WNT/ERK pathways and HDAC inhibition (Liang et al., 2022).

## 6 Gut microbiota mechanisms in allergic asthma

**FIGURE 2:**
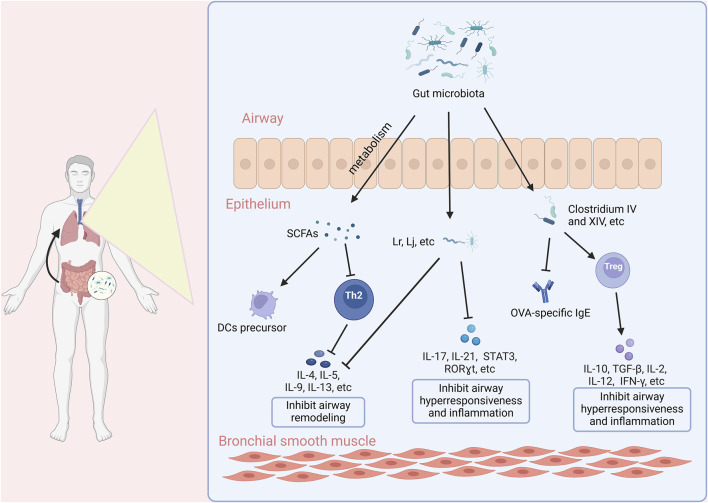
The relationship between gut microbiota and allergic asthma. SCFAs, Short-chain fatty acids; DCs, Dendritic Cells.

We delineate the multifaceted role of gut microbiota in allergic asthma pathophysiology, highlighting microbial-immune interactions as a therapeutic frontier for airway hyperreactivity, remodeling, and allergenic sensitization as shown in Figure 2.

### 6.1 Airway hyperresponsiveness and microbial regulation

The nexus between gut microbiota imbalance and airway hyperresponsiveness (AHR) has emerged as a pivotal focus in asthma pathogenesis. AHR, a hallmark of allergic asthma, is intricately linked to gut microbial composition and diversity through immune crosstalk. Gut microbiota modulate airway inflammation via systemic immune regulation, with microbial metabolites like short-chain fatty acids (SCFAs) playing central roles. Liu et al. demonstrated that SCFAs suppress inflammatory cytokine release, directly attenuating AHR severity (Liu et al., 2023). Patients with AHR exhibit marked reductions in gut microbial diversity, particularly depletion of immunomodulatory taxa such as *Bifidobacterium* and *Lactobacillus*, which may compromise immune tolerance and exacerbate allergic sensitization (Chen et al., 2024). Intervention studies highlight therapeutic potential: *Lactobacillus johnsonii* supplementation reduces immune cell activation in lungs and Th2 cytokine expression (IL-4, IL-5, IL-13, IL-17), suggesting microbiota-targeted strategies for AHR modulation (Aagaard et al., 2012).

### 6.2 Gut microbiota and airway remodeling

Airway remodeling—characterized by structural alterations like smooth muscle hypertrophy and subepithelial fibrosis—is mechanistically influenced by gut microbiota via systemic inflammatory pathways. Chen et al. identified that specific gut microbes inhibit remodeling-associated cytokines (IL-4, IL-5, IL-9, IL-13) through SCFA production, mitigating airway wall thickening (Chen et al., 2024). Microbial imbalance may potentiate smooth muscle hyperplasia by enhancing pro-fibrotic signaling, as evidenced in murine models where *Lactobacillus rhamnosus* (Lr) administration reduced leukocyte infiltration, bronchial hyperreactivity, and remodeling markers (IL-4, IL-5, IL-13, STAT6, GATA3, IL-17, IL-21, IL-22, STAT3, RORγt) in asthma-COPD overlap syndrome (Vasconcelos et al., 2023). These findings position microbiota modulation as a viable approach to attenuate or reverse remodeling processes, potentially improving long-term asthma outcomes.

### 6.3 Microbial modulation of allergic sensitization

Gut microbiota critically shapes immune responses to allergens through T cell polarization and cytokine regulation. Pantazi et al. revealed that select commensals enhance regulatory T cell (Treg) differentiation, suppressing allergic effector responses (Pantazi et al., 2023). Murine studies demonstrate that *Clostridia* clusters IV/XIV supplementation elevates colonic Tregs and IL-10 production, correlating with reduced ovalbumin-specific IgE and IL-4 levels in allergic models (Atarashi et al., 2011). In allergic asthma patients, microbial imbalance disrupts this immunoregulatory balance, amplifying hypersensitivity to aeroallergens (e.g., dust mites, pollen) and triggering exacerbations (Han et al., 2024). Microbial metabolites, particularly SCFAs, further mitigate allergic sensitization by modulating DC function and Th2 cytokine production. These mechanistic insights underscore the potential of microbiota-targeted interventions—probiotic supplementation, dietary modulation, or metabolite administration—as novel strategies for asthma prevention and management (Du et al., 2022; Zheng et al., 2023).

### 6.4 Gut-lung axis in asthma pathogenesis

SCFAs (e.g., butyrate) circulate to the lung, activating GPR43 on Tregs to suppress Th2-mediated eosinophilia (Yao et al., 2022). Tryptophan metabolites (e.g., IPA) activate pulmonary AhR, inducing IL-22 production by ILC3s (Zhang et al., 2024). Gut-primed DCs migrate to the lung via lymphatics, regulating local Th17/Treg balance (Zhang et al., 2022). Microbial imbalance reduces vagal tone, impairing acetylcholine-dependent suppression of airway mast cell degranulation (de Haan et al., 2013). Clinical evidence linking depleted Faecalibacterium prausnitzii (and reduced SCFAs) to airway hyperresponsiveness, reversible via probiotics, is also included (Hu et al., 2021b).

## 7 Intervention strategy based on gut microbiota

Probiotics, defined as live microorganisms conferring host health benefits, modulate gut microbial equilibrium by enhancing colonization resistance, immunomodulation, and epithelial barrier reinforcement. Clinical applications span gastrointestinal disorders (diarrhea, constipation, inflammatory bowel disease) through mechanisms involving pathogen exclusion, bacteriocin production, and immune cell priming (Li et al., 2024). Prebiotics—non-digestible substrates selectively fermented by commensals—stimulate beneficial taxa proliferation (e.g., *Bifidobacterium*, *Lactobacillus*) while increasing short-chain fatty acid (SCFA) production, thereby improving intestinal homeostasis (You et al., 2022). Synbiotic formulations combining probiotics with prebiotics demonstrate synergistic effects, enhancing microbial diversity and metabolic functions more effectively than individual components (Du et al., 2024). Despite therapeutic promise, clinical translation requires rigorous validation through randomized controlled trials to establish strain-specific mechanisms, dosing protocols, and long-term safety profiles (Raghani et al., 2024). Special attention should be paid to leveraging strain specific effects, such as lactobacilli competing with pathogenic bacteria for nutrients and adhesion sites by occupying space on the surface of the intestinal mucosa, thereby inhibiting the overgrowth of harmful bacteria.

Dietary patterns exert profound effects on gut microbiota composition and functionality (Leeming et al., 2019). High-fiber diets increase SCFA producers by ∼40% (*Faecalibacterium*, *Roseburia*) and improving metabolic parameters through GLP-1 secretion and hepatic gluconeogenesis suppression (Fu et al., 2022). Conversely, Western-style diets high in saturated fats and refined sugars drive microbial imbalance, characterized by *Bacteroides* enrichment and reduced microbial diversity, correlating with chronic inflammation and metabolic syndrome (Mehmood et al., 2021). Temporal dynamics further influence intervention efficacy: transient dietary changes induce reversible microbial shifts, whereas sustained dietary habits remodel enterotypes, suggesting long-term adherence is critical for durable ecological benefits (Li et al., 2024). Precision nutrition strategies, integrating host genetics, microbiota profiling, and lifestyle factors, represent emerging paradigms for personalized microbiota engineering.

Advancements in microbiota research are driving the development of multidimensional intervention frameworks. Combinatorial approaches—integrating probiotics, prebiotics, dietary modifications, and phage therapy—show enhanced efficacy in restoring microbial networks disrupted in conditions like obesity and diabetes (Sumida et al., 2023). Elucidating cross-system interactions (e.g., microbiota-immune-nervous axis crosstalk) will uncover novel therapeutic targets, as evidenced by SCFA-mediated neuroimmune regulation in allergic airway diseases [54]. Clinically, microbiota-targeted therapies are being incorporated into disease-specific protocols, including fecal microbiota transplantation for *C. difficile* infection and engineered probiotics for inflammatory bowel disease (Xu et al., 2024). However, challenges persist in standardizing microbial products, optimizing personalized dosing, and establishing long-term safety monitoring systems. Moreover, the optimal dosage and long-term safety of probiotics have not yet been determined. Addressing these barriers will be pivotal for translating microbiota science into mainstream clinical practice, ultimately enabling precision medicine approaches for complex chronic diseases.

## 8 Conclusion

New evidence highlights the key role of gut microbiota and their metabolites in allergic asthma development. Microbial imbalance—altered diversity, changes in key taxa (e.g., bifidobacteria) and metabolites (e.g., SCFAs, tryptophan derivatives)—may link to immune dysfunction. These microbe-immune interactions improve our understanding of asthma and reveal new microbial-targeted therapies. Probiotics, prebiotics, and dietary changes show potential to reset immune responses and reduce asthma severity. Yet, while microbial shifts correlate with disease, causal links are poorly defined, requiring more mechanistic research using gnotobiotic models and long-term human studies.

Future research should focus on clarifying strain-specific microbial functions, host-microbe interaction pathways (e.g., gut-lung axis signaling), and individual responses influenced by genetics, environment, and diet. Validating strain-specific probiotics through human trials is crucial. Large-scale multi-omics cohorts combined with randomized controlled trials of targeted microbiota interventions are needed to confirm therapeutic effects and improve precision medicine approaches. In short, the gut microbiota is both a biomarker and a modifiable driver of allergic asthma. Unraveling its complex interactions with host immunity and physiology will advance microbiome-based strategies for asthma prevention, personalized treatment, and long-term control.
